# Characterization of bacterial microbiota compositions along the intestinal tract in pigs and their interactions and functions

**DOI:** 10.1038/s41598-018-30932-6

**Published:** 2018-08-24

**Authors:** Daniel Crespo-Piazuelo, Jordi Estellé, Manuel Revilla, Lourdes Criado-Mesas, Yuliaxis Ramayo-Caldas, Cristina Óvilo, Ana I. Fernández, Maria Ballester, Josep M. Folch

**Affiliations:** 1 Plant and Animal Genomics, Centre for Research in Agricultural Genomics (CRAG), CSIC-IRTA-UAB-UB Consortium, Bellaterra, Spain; 2 Departament de Ciència Animal i dels Aliments, Facultat de Veterinària, Universitat Autònoma de Barcelona (UAB), Bellaterra, Spain; 3 Génétique Animale et Biologie Intégrative (GABI), Institut National de la Recherche Agronomique (INRA), AgroParisTech, Université Paris-Saclay, Jouy-en-Josas, France; 4 Departament de Genètica i Millora Animal, Institut de Recerca i Tecnologia Agroalimentàries (IRTA), Caldes de Montbui, Spain; 5 Departamento de Mejora Genética Animal, Instituto Nacional de Investigación y Tecnología Agraria y Alimentaria (INIA), Madrid, Spain

## Abstract

In addition to its value in meat production, the pig is an interesting animal model for human digestive tract studies due to its physiological similarities. The aim of this study was to describe the microbiome composition, distribution and interaction along the Iberian pig intestinal tract and its role in whole-body energy homeostasis. The V3-V4 region of the 16S rRNA gene was amplified and sequenced from the microbiomes of five gut sections (duodenum, jejunum, ileum, and proximal and distal colon) in thirteen castrated male pigs. A total of 1,669 operational taxonomic units distributed in 179 genera were found among all samples. The two most abundant genera in the small intestine were *Lactobacillus* and *Clostridium*, while *Prevotella* was predominant in the colon. The colon samples were more similar among the pigs and richer in species than the small intestine samples were. In the small intestine, the metagenome prediction pointed to rapid internalization and conversion of the available simple carbohydrates for microbial proliferation and maintenance. In the colon, a competition among anaerobic bacteria for plant polysaccharide degradation to produce short chain fatty acids was found. This study confirms that the energy pathways of the gut microbiome differ along its sections and provides a description of the correlations between genera.

## Introduction

Trillions of microbes colonize the mammalian intestinal tract, supplying functions that in most cases the host cannot perform, such as digesting vegetable fibre and harvesting energy from otherwise inaccessible nutrients^1^. In humans, the genomes of these microorganisms (the so-called metagenome) contain more than 9 million unique genes^2^. With the publication of the first reference gene catalogue of the porcine gut microbiome^3^, made through shotgun metagenome sequencing, the number of non-redundant genes identified reached 7.7 million in pigs. In addition to its interest for meat production, the pig is used as an animal model for human research due to the similarity in digestive tract anatomy, physiology, and immunology between pigs and humans^4^. Furthermore, both species share more non-redundant genes in their microbiota than humans do with other model organisms, such as the mouse^3^. Nevertheless, much of the work on the relationship between human obesity and the gut microbiota has been performed in mice^5^.

Beyond whole-metagenome sequencing, an alternative cost-effective approach to studying the microbiota relies on targeted re-sequencing of the variable regions of the microbial 16S rRNA gene^6,7^. In recent years, the number of publications analysing the pig gut microbiota with the 16S approach has increased exponentially^8–14^. Interestingly, the pig gut microbiota composition has recently been related to average daily weight gain and to body weight^10,13^, feed efficiency^15^, feed conversion and feed intake^16^. However, only a few studies have analysed in detail the microbiota profiles in different parts of the digestive tract^17–19^. While these studies were focused on microbiota analysis along the digestive tract of the Large White^17^, Laiwu^18^ and Gloucestershire Old Spot^19^ breeds, the microbiota profile of the Iberian pig and the correlations between genera along the pig gut have not yet been described.

The Iberian pig is a rustic animal with a higher adipogenic trend and lower meat efficiency than those of commercial breeds. Its high intramuscular fat content and backfat thickness are optimal for ham production. This excellent organoleptic quality is due to its high fat infiltration rate, with a high proportion of oleic fatty acid (C18:1(n-9)), along with a smaller proportion of polyunsaturated fatty acids^20^. In this context, Bäckhed *et al*.^21^ found that, in mice, lipid metabolism can be modified by the gut microbiota. Therefore, unveiling the microbiota composition in Iberian pigs and how it varies along the digestive tract may provide a basis for understanding the effects of the microbiome on lipid metabolism in pigs.

The aim of this study was to describe the interactions and differences in the microbiome found along the Iberian pig gut and to evaluate their possible role in whole-body energy homeostasis. To this end, we explored the pig microbiota composition in five gut sections (duodenum, jejunum, ileum, and proximal and distal colon) by 16S rRNA gene sequencing.

## Results

### Microbial taxonomic composition shows major differences along the intestine

**Figure 1:**
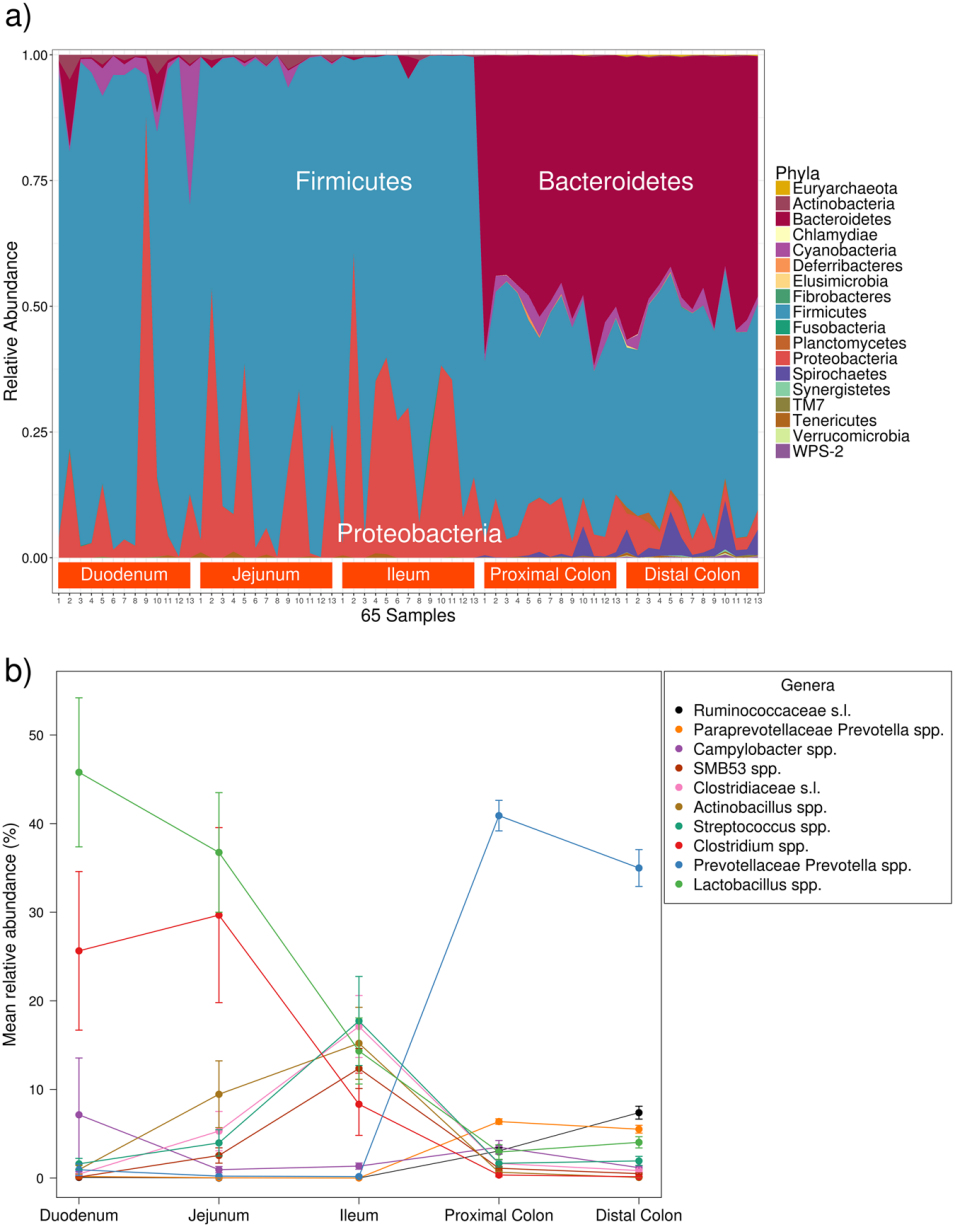
(**a**) Stacked area plot of the OTUs grouped by phyla for the 65 samples sorted by intestinal section. (**b**) Percentage evolution along the gut of the ten most abundant bacterial genera in the dataset. Segments represent the standard error.

The V3-V4 region of the 16S rRNA was amplified and sequenced from the luminal contents of five gut sections (duodenum, jejunum, ileum, and proximal and distal colon) of thirteen Iberian pigs aged 120 days. A *MiSeq*^®^ (*Illumina*^®^) instrument was used to obtain a mean of 126,549 sequences per sample. The sequences were processed and filtered through the QIIME pipeline^22^, and a total of 1,669 operational taxonomic units (OTUs) were obtained among the five sections. According to the Greengenes 13.8 database^23^, 643 were new OTUs. In addition, the 1,669 OTUs were aggregated into 179 genera and 18 phyla (Fig. 1a). The two most abundant genera in the duodenum and jejunum were *Lactobacillus* (45.79% and 36.75%, respectively) and *Clostridium* (25.64% and 29.67%, respectively) (Fig. 1b). Conversely, the two most abundant genera in the ileum were *Streptococcus* (17.73%) and the unspecified genera of the Clostridiaceae family (17.10%). Other genera of the Clostridiaceae family had a modest abundance in the ileum: *SMB53* (12.36%) and *Clostridium* (8.33%). The *Prevotella* genus was the most dominant in the colon, representing 40.90% in the proximal part and 34.99% in the distal one.

**Figure 2:**
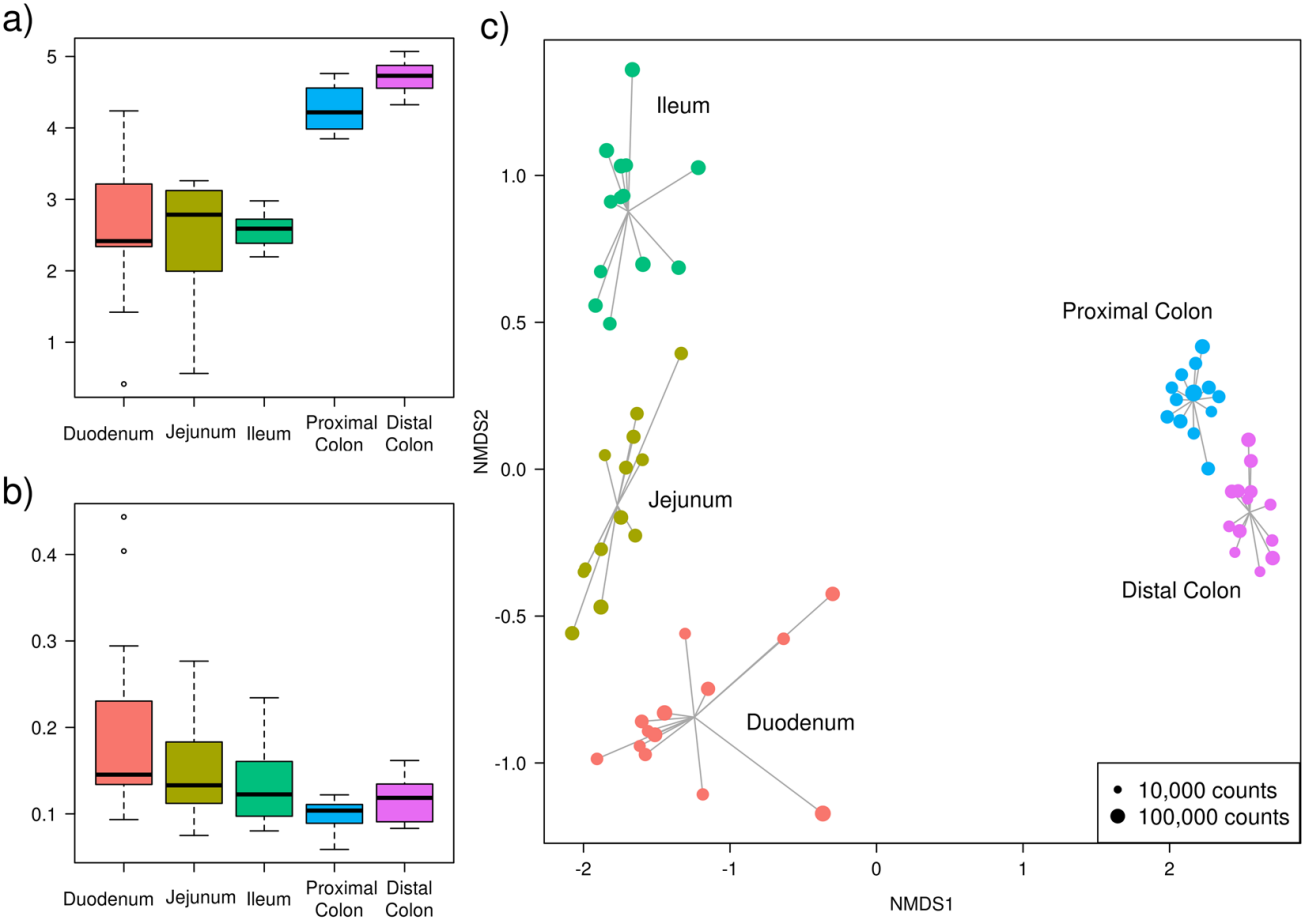
Descriptive plots made from the OTUs obtained in each sample. (**a**) Boxplot of the Shannon α-diversity for the 13 pigs in each intestinal section. (**b**) Boxplot of the Whittaker β-diversity for the 13 pigs in each intestinal section. (**c**) Non-metric multidimensional scaling (NMDS) plot based on Bray-Curtis dissimilarities for the 65 samples of the 13 pigs in each of the 5 intestinal sections (represented by colours). The size of the dot is proportional to the total number of counts in each sample, as represented in the bottom-right rectangle.

Shannon index was used to evaluate the community α-diversity for each sample. The Shannon diversity measures the number of different species and their relative abundance within a sample. The α-diversity was higher in the large intestine than in the small intestine sections (Fig. 2a). In fact, the small intestine samples showed a larger variation in α-diversity values among individuals, whereas the colon samples had higher and more constant values. On another level, the β-diversity measures the differences between samples. These differences are shown in Fig. 2b, where the average pairwise distances of each group of samples to the group centroid (β-diversities) were obtained with the Whittaker index calculated through Bray-Curtis dissimilarities. In contrast with the α-diversity, which increased along the gut sections from the duodenum to the distal colon, the β-diversity decreased. Hence, there was a higher similarity among large intestine samples despite their high α-diversity, while small intestine regions such as the duodenum showed higher differences among individuals. These dissimilarities were observed in the non-metric multidimensional scaling (NMDS) plot, where samples were consistently separated according to their intestinal section (Fig. 2c). Indeed, a large distance between the small and large intestines is depicted. The points representing the colon samples are closer to their centroid than those of the small intestine, in accordance with the β-diversity analysis (Fig. 2b).

**Figure 3:**
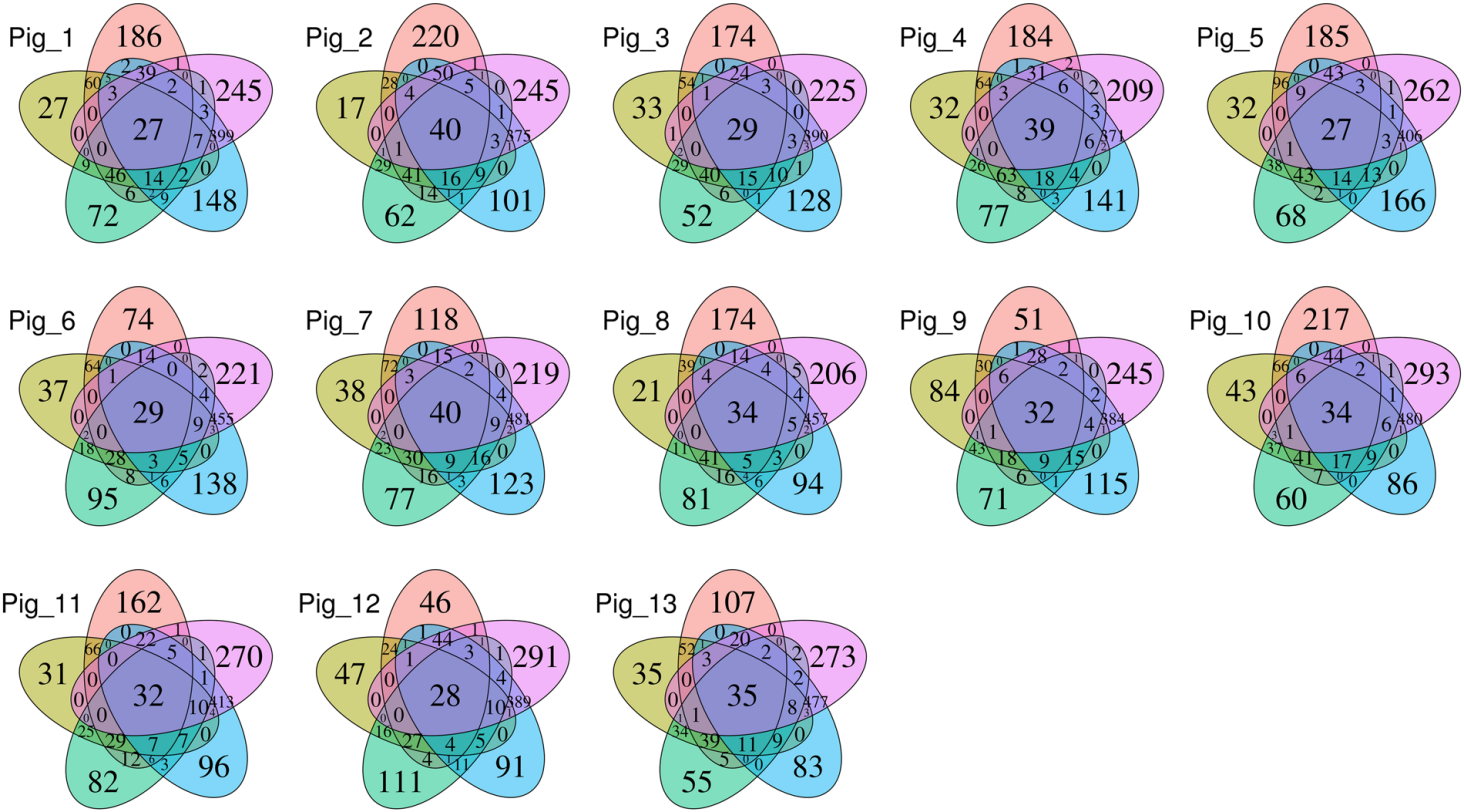
Five-part Venn diagram for each of the 13 subjects, showing the OTUs shared among the intestinal sections: duodenum (red), jejunum (yellow), ileum (green), proximal colon (blue), and distal colon (purple). The numbers in the diagrams represent how many OTUs were unique in the five intestinal sections or shared between sections as their areas intersect.

To describe which OTUs were present in all animals along all sections, that is, the minimum core microbiota, an OTU was assumed to be present if it had at least one count in a given sample (Fig. 3). It is noticeable that the greatest number of OTUs were detected in only the distal colon samples. Moreover, the intersection between the two colonic regions had the highest number of OTUs. In contrast, the jejunum was the gut segment with the lowest number of unique OTUs. The duodenum, in accordance with its high β-diversity, also had a high number of unique OTUs in each sample. The intersection between the duodenum and the two large intestine regions shared a mean of 29.8 OTUs. However, the intersection between the duodenum and the other two small intestine sections shared a slightly greater number of OTUs (mean of 37.4). In addition, the number of OTUs shared among all the intestinal regions was between 27 and 40 for each animal. Nevertheless, when combining the datasets from all animals, 44 of the 1,669 OTUs were shared among the five intestinal regions, representing 71% of the total number of counts (see Supplementary Fig. S1). In the core formed by these 44 OTUs, only two were new OTUs, and the most abundant genera were *Lactobacillus* with 13 OTUs (23.74% of the core) and *Clostridium* with one OTU (22.45% of the core). Additionally, from the 1,669 total OTUs, 946 were absent in the small intestine sections, whilst 325 were not present in the large intestine (see Supplementary Fig. S1).

### Microbiota interaction network reconstruction among the gut sections

**Figure 4:**
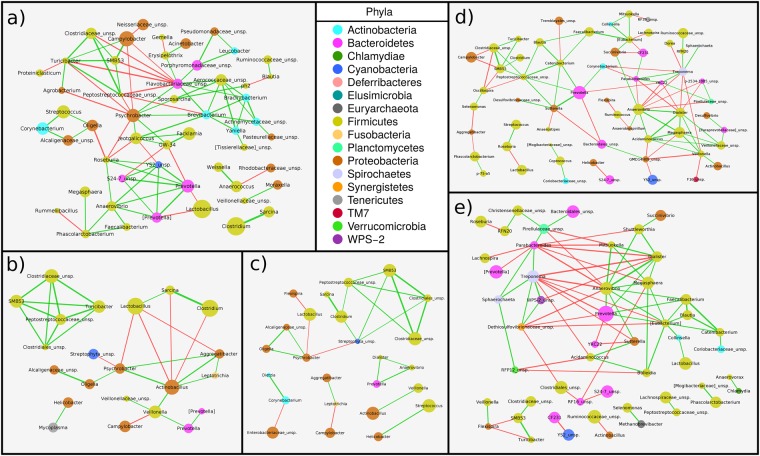
SparCC^55^ PCIT^56^ (SPCIT^13^) prokaryotic genus network for partial correlations with an absolute value above 0.65 between log-transformed genus abundances performed in the (**a**) duodenum, (**b**) jejunum, (**c**) ileum, (**d**) proximal colon, and (**e**) distal colon. The width of the edge represents the degree of the correlation (wider if it is higher), and the colour shows the sign of the correlation: negative (red) and positive (green). The area of the node is proportional to the relative abundance of the prokaryotic genus. The suggested annotation for some genera is enclosed in square brackets.

To infer the interaction patterns as well as the hub genera in each section, microbial interaction networks were calculated for each gut section by using the SPCIT method as proposed by Ramayo-Caldas *et al*.^13^. For this network analysis, the correlations between genera were estimated using the relative abundances of each genus across the animals (n = 13) in each of the 5 intestinal segments. In the network, every node represents a genus, and every edge connecting two nodes represents a SPCIT significant correlation: only those correlations above an absolute value of 0.65 were represented (Fig. 4).

In the duodenum (Fig. 4a), *Psychrobacter* spp., *Jeotgalicoccus* spp. and the unspecified Flavobacteriaceae genera were part of a sub-network showing a negative correlation with another sub-network formed by *Campylobacter* spp. and *SMB53* spp. The most abundant genus in the duodenum was *Lactobacillus*, which was negatively correlated with *Prevotella* spp. The other two most abundant genera were *Clostridium* and *Sarcina*, both belonging to the Clostridiales order and showing a strong positive correlation between them, which was maintained along all the small intestine until the disappearance of *Sarcina* spp. in the hindgut.

Regarding the jejunum (Fig. 4b), *Lactobacillus* spp. were associated with the two strongly linked Clostridiales genera *Clostridium* and *Sarcina*. Conversely, the *Actinobacillus* and *Psychrobacter* genera were negatively correlated to this sub-network. However, the rest of the Clostridiales order were correlated only among themselves.

In the ileum (Fig. 4c), the strong correlation within the Clostridiales order, including members such as the paired *Clostridium*-*Sarcina* genera, was maintained. It should be noted that *Lactobacillus* spp. were still negatively correlated with *Psychrobacter* spp. Moreover, in spite of the abundance of the *Actinobacillus* and *Streptococcus* genera, they were correlated only with *Veillonella* spp. and *Helicobacter* spp., respectively.

The number of significant correlations increased in the proximal colon (Fig. 4d), where the most abundant *Prevotella* spp. took the central role. *Prevotella* spp. were strongly positively correlated with *Sutterella* spp., and both of them with the Clostridiales order group. In contrast, *Campylobacter* spp. were negatively correlated with the Clostridiales order group. In addition, *Prevotella* spp. showed a negative correlation with the sub-network formed by *Treponema* spp. and *Parabacteroides* spp. This last sub-network was also in opposition to the one formed by the *Anaerovibrio*, *Dialister*, and *Megasphaera* genera.

In the last section, the distal colon (Fig. 4e), *Prevotella* spp. were positively correlated with the sub-network of the *Anaerovibrio*, *Dialister*, and *Megasphaera* genera, which were negatively correlated with the *Treponema* and *Parabacteroides* sub-network, as observed in the proximal colon. *Lactobacillus* spp. were likely to be in the same group as *Prevotella* spp., and the Clostridiales order group was not clearly correlated with the other sub-networks.

### Presence/absence and differential abundance analysis of genera between consecutive sections

To compare the relative abundances of genera between the sections, we analysed each pair of consecutive regions by using metagenomeSeq^24^. After determining the presence of the genera, a differential abundance analysis was performed to filter out any genera that were absent in either of the two compared regions. The four comparisons of the two types of tests (presence/absence and differential abundance) are shown in the supplementary information found online as Supplementary Tables S1 and S2, respectively.

Regarding the first comparison, duodenum versus jejunum, it was clearly observed that the duodenum contained more unique genera than the jejunum (38 against two). In the duodenum, there were several genera of the Actinomycetales, Bacillales, and Clostridiales orders not present in the jejunum, while *Actinomyces* spp. and *Catenibacterium* spp. were present in only the jejunum. However, there were few differentially abundant genera in this comparison (five in the duodenum and seven in the jejunum). The five most abundant bacterial genera in the duodenum were distributed in phyla as follows: one Cyanobacteria, two Firmicutes (both from the Lactobacillales order) and two Proteobacteria (belonging to the Moraxellaceae family). In contrast, the jejunum had more Firmicutes (*Turicibacter* spp. and three Clostridiales) and three Proteobacteria (*Helicobacter* spp., *Actinobacillus* spp. and an unspecified Enterobacteriaceae genus).

For the jejunum versus ileum comparison, the jejunum retained the two specific genera (*Actinomyces* and *Catenibacterium*) observed in the previous comparison, as well as two additional taxa, Bacteroidetes and *Blautia* spp., as differentially present. On the other hand, only one genus (*Flexispira* spp.) was differentially present in the ileum when compared with the jejunum. In the differential abundance study, only the Cyanobacteria phylum, which was previously more abundant in the duodenum, was shown to have a higher abundance in the jejunum than in the ileum. The eight genera that were more abundant in the ileum include four Clostridiales and other genera such as *Actinobacillus* and *Streptococcus*.

As expected from the great distance observed in the NMDS plot between the small and large intestine samples, the comparison between the ileum and proximal colon showed the highest divergence, with a total of 64 genera being differentially present in both sections. Of these genera, only eight were differentially present in the ileum, and the rest of them (56) were in the proximal colon. The Cyanobacteria phylum was present in only the ileum when comparing it with the proximal colon, as well as other genera, such as *Dietzia*, *Facklamia* and *Sarcina*. In contrast, of the 56 genera differentially present in the proximal colon, 26 were from the Firmicutes phylum (20 from the Clostridiales order including *Butyrivibrio* spp., *Roseburia* spp. and *Ruminococcus* spp., and 6 from the Erysipelotrichales order). From the remaining 30 genera differentially present in the proximal colon, 9 belonged to the Bacteroidetes phylum, 9 were members of the Proteobacteria phylum, one was a species of Archaea from the *Methanobrevibacter* genus, and the other 11 included genera such as *Treponema* and *Chlamydia*. However, the number of differentially abundant genera between the two sections decreased to 22, with 15 genera significantly more abundant in the ileum: 8 Firmicutes (five Clostridia, including the *Veillonella* and *Clostridium* genera, and three Bacilli, *Lactobacillus* spp., *Streptococcus* spp. and *Turicibacter* spp.), 5 Proteobacteria (including *Actinobacillus* spp., *Psychrobacter* spp. and *Flexispira* spp.), and *Corynebacterium* and *Mycoplasma*. In contrast, seven genera were more abundant in the proximal colon, six of them belonging to the Clostridiales order (5 inside the Veillonellaceae family), and one from the Bacteroidales order (*Prevotella* spp.).

It is noteworthy that no genus was found to be differentially present in the proximal colon when compared with the distal colon. However, nine genera were present in only the distal colon: *Fibrobacter* spp., *Anaerovorax* spp. and two Archaea related to methane metabolism, among others. Additionally, the differential abundance study between these hindgut sections pointed out 15 genera with higher abundance in the proximal colon: one genus of the Cyanobacteria phylum, one from the Deferribacteres phylum, and seven Clostridiales genera from the Clostridiaceae and Veillonellaceae families, as well as six genera from the Proteobacteria phylum (including *Campylobacter* spp., *Helicobacter* spp. and *Actinobacillus* spp.). In the distal colon, of the 16 differentially abundant genera in comparison to the proximal colon, 5 belonged to the Bacteroidales order, 4 were Clostridiales, and from the rest, *Treponema* spp. and a species of Archaea (*Methanobrevibacter* spp.) stood out.

### Functional analysis of the gut metagenome along the intestine

**Figure 5:**
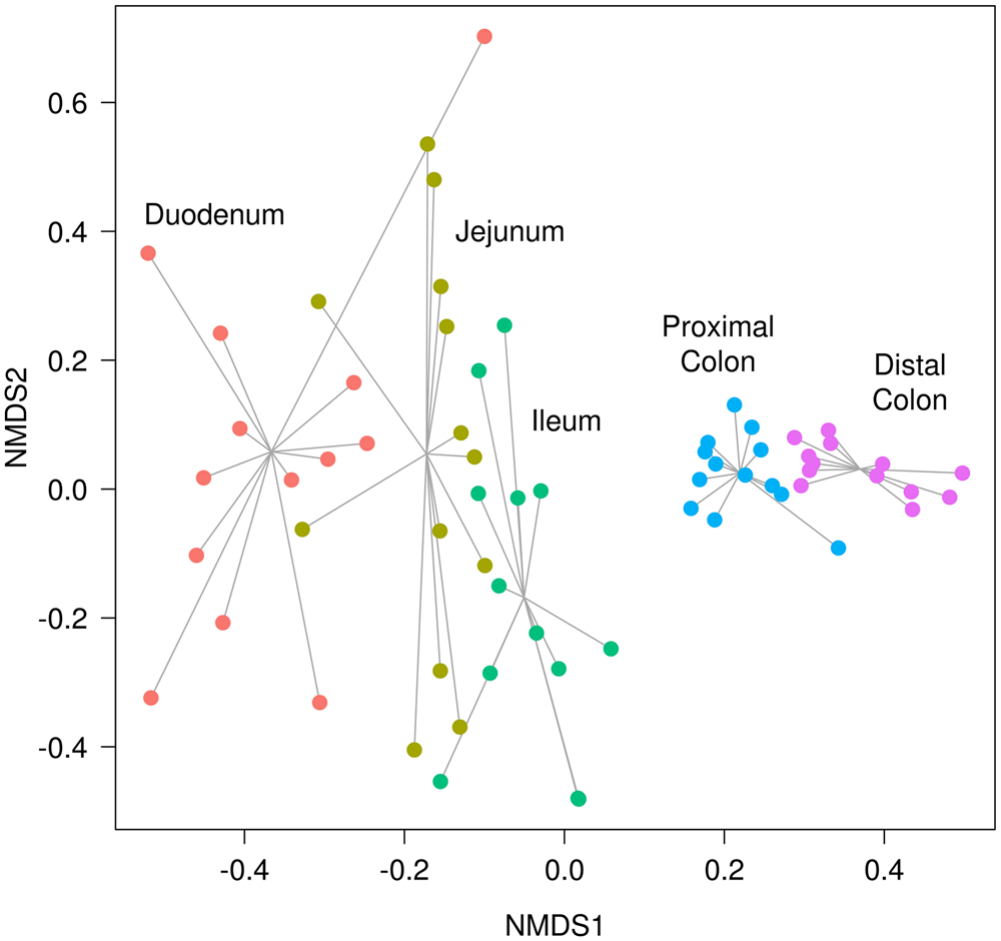
Non-metric multidimensional scaling (NMDS) plot based on Bray-Curtis dissimilarities for the metagenome (KEGG^26^ orthologies (KOs) counts) predicted through PICRUSt^25^ for the 65 samples from the 13 pigs in each of the 5 intestinal sections (represented by colours). In this plot, it can be seen how the predicted functions for the microbiota of the large intestine sections are more similar among individual pigs, while the predicted functions for the microbiota of the small intestine sections have more variation among individual pigs, meaning that large intestinal microbiotas are more likely to perform similar functions.

Finally, PICRUSt^25^ was used for metagenomic functional prediction of each of the five regions. PICRUSt utilizes 16S rRNA gene information to estimate the gene families of Archaea and bacteria that contribute to a metagenome. An NMDS analysis was performed to determine whether sample distribution depended on the predicted KEGG^26^ orthologies (KOs) table (Fig. 5). This plot depicts how the separation between the small and large intestines was still clearly maintained (from left to right in the plot). In addition, the proximity of the hindgut samples reveals that they are more likely to perform the same functions, which is in accordance with the similarities found in their microbiota compositions with respect to β-diversity (Fig. 2b). Conversely, this NMDS plot (Fig. 5), made from the predicted KOs, showed that some of the small intestine samples of one section (e.g., jejunum) were closer to samples collected from other small intestine sections (e.g., duodenum and ileum) than to samples from their own section (e.g., jejunum).

To gain a better understanding of the differentially abundant functions between the regions, the KOs were collapsed to the pathway level. Then, DESeq2^27^ was utilized to compare the collapsed pathway abundances between each pair of consecutive regions; a pathway was considered more abundant in one section if its adjusted *p*-value was ≤ 0.01. The results are shown in the supplementary information found online as Supplementary Table S3.

In the first comparison, duodenum versus jejunum, a clear enrichment for the carotenoid and flavonoid biosynthesis pathways in the duodenum was found. In addition, the antenna proteins of the photosynthesis pathway were also more abundant in the duodenum. Nevertheless, the most abundant pathway found in the duodenum was N-glycan biosynthesis. In contrast, the transporters and energy metabolism pathways, including fructose, mannose, amino sugar and nucleotide sugar metabolism, were more abundant in the jejunum.

In the comparison between the jejunum and ileum, pathways related to photosynthesis were still abundant in the jejunum. The most abundant pathways in the jejunum compared with the ileum were the basal transcription factors and pathways related to mineral absorption and glycolysis/gluconeogenesis and, to a lesser extent, bile acid biosynthesis and lysosomes. Conversely, the most abundant pathways in the ileum were tetracycline and polyketide sugar unit biosynthesis, both related to antibiotic synthesis. To a lesser degree, the fatty acid and lipid protein biosynthesis pathways stood out, as well as those related to amino acid metabolism.

The largest number of significant differences was found between the ileum and the proximal colon. In the ileum, pathways related to transporters and transcription factors were more abundant than in the proximal colon. The tetracycline- and lipid-related pathways were maintained in the ileum, as in the previous comparison. In addition, some degradation pathways (dioxin, ketone bodies, benzoate and xylene) were also abundant in the ileum, as well as the metabolism of pyruvate and two short chain fatty acids (SCFAs), propanoate and butanoate. Finally, one of the most relevant abundant pathways in the ileum was the phosphotransferase system, which is a bacterial method of sugar uptake. In the opposite direction, in the proximal colon, the most abundant functions were carbon fixation in photosynthetic organisms, biosynthesis of vancomycin group antibiotics, and protein digestion and absorption. Additional functions more abundant in the proximal colon were the adipocytokine and PPAR signalling pathways; sphingolipid, arachidonic acid, beta-alanine and vitamin B6 metabolism; phenylalanine, tyrosine and tryptophan biosynthesis; and, similar to in the jejunum, mineral absorption and lysosomes. Lastly, it is also worth mentioning the carbon fixation pathways in prokaryotes as well as oxidative phosphorylation and the citrate cycle (Krebs cycle).

In the last comparison between the two hindgut sections, the proximal and distal colon, the most abundant differential functions in the proximal colon were the lipopolysaccharide and its protein biosynthesis; metabolism of cofactors and vitamins, such as folate and riboflavin, was also notable, as well as glutathione and arachidonic acid metabolism. However, in the distal colon, the pathways related to bacterial cell walls were significantly more abundant (pentose and glucoronate interconversions), as well as two pathways associated with amoebiasis and bacterial antibiotic production (butirosin and neomycin biosynthesis). Nevertheless, other functions related to carbohydrate, pyruvate and methane metabolism were also identified.

The methane metabolism pathway was analysed in detail at the KO level for each comparison (see Supplementary Fig. S2), and the results showed that methane production was more abundant in the distal colon and acetate production was significantly greater in the hindgut.

## Discussion

This study characterized the composition, distribution and potential functionality of the microbiota found in the luminal content of five sections along the digestive tract of 13 Iberian pigs at 120 days of age fed with a maize- and wheat-based diet. We confirmed the existence of extensive differences in the microbiota composition along the porcine intestine, especially between the small and large intestines, and we provide additional insights on the ecosystem structure in each section and its potential functional consequences.

In the midgut (duodenum, jejunum, and ileum) we observed a low α-diversity and a high β-diversity, while the large bowel (proximal and distal colon) had a high α-diversity and a low β-diversity. The lower α-diversity (Fig. 2a) of the midgut compared with the higher α-diversity of the large bowel found in our samples was previously described in the luminal contents of 300-day-old Laiwu pigs when comparing three sections (jejunum, ileum and caecum)^18^. These differences in diversity between the small and large intestines were also found in another study of the mucosa microbiota of 28-day-old pigs^19^ from the Gloucestershire Old Spot breed. In addition, the β-diversity (Fig. 2b) analysis pointed out that the differences between samples in the small intestine region were higher than those in the colon. This reduction in β-diversity when descending through the gut was also described in Laiwu pigs by Yang *et al*.^18^, and it can be seen in our results, both in the NMDS (Fig. 2c), where the points are closer in the hindgut, and in the smaller standard errors represented in the ten most abundant genera plot (Fig. 1b). On the other hand, the greatest β-diversity was observed in the duodenum samples, with two samples very differentiated from the rest. One of these is the same outlier that appears in the α-diversity plot (Fig. 2a), and thus, this individual could have a reduced diversity due to some kind of asymptomatic disease.

The higher variability observed between the midgut samples may be due to the lower number of microorganisms present in these regions. Thus, the bacterial community could potentially be less stable in the midgut than in the large bowel sections because of the continuous influx of new bacteria from food, the shorter transit time and the importance of adherence to tissue or mucus^28^. Moreover, the bacteria in the small intestine may be more susceptible to a founder effect: when early colonizers arrive, they are more prone to become established and provide the nutrients to establish a certain microbiota, but the mechanisms of this effect remain unknown^28^. Nonetheless, these pigs were raised and fed together, being exposed to similar environmental conditions; therefore, the greater differences between the small intestine samples may be due to host genetic factors, such as those related to the immune responses of the animals^29^.

Consistent with the differences between intestinal sections and in accordance with other studies of the luminal^17,18^ and mucosal^19^ microbiota in pigs, the three most abundant phyla found along the digestive tract were Bacteroidetes, Firmicutes and Proteobacteria (Fig. 1a). Nevertheless, comparing one study of the luminal microbiota with another study of the mucosal microbiota is complicated. The intestinal epithelium provides an oxygen-rich environment that could be very different from that of the luminal content^30,31^, differentiating the microbiota found in these two regions^19^. Furthermore, the ratios of species abundance obtained in the two luminal content studies are difficult to compare because several variables are present in the experiments: 6-month-old Large White pigs fed with a standard diet based on maize^17^ and 300-day-old Laiwu pigs fed with a maize-soybean diet^18^. In addition, it has been shown that different ages^17^, feeding^32^, and DNA extraction kits^33^, amongst other factors, can have an impact on the observed microbiota composition. In this sense, the breed or the genetic background of the pigs might alter the microbiota composition as well^16,34^. For example, despite the limitations of comparing two studies, Zhao *et al*.^17^ described in the colon of Large White pigs a low proportion of Bacteroidetes (8.5%), while we found that the *Prevotella* genus, which belongs to the Bacteroidetes phylum, represented almost 41% in the proximal colon and 35% in the distal colon of the genera found in the Iberian pig. These two breeds are very different in lipid content; the Iberian pig is characterized by a high fat infiltration rate^20^, whereas the Large White pig produces leaner meat (with a lower fat content). Hence, these differences in lipid content might be due to bacteria such as *Prevotella* spp. that can degrade the proteins and polysaccharides in the plant cell wall, producing SCFAs that can be absorbed by the host^8,35^ and can modify the host lipid metabolism, increasing fat retention and adipogenesis^36,37^. Further studies are required to validate such differences in microbiota composition between breeds and their possible relationships with fat metabolism.

In our study, the Bacteroidetes and Firmicutes dominated in the colon, and the Firmicutes phylum was also the most abundant in the small intestine. The presence of the Proteobacteria phylum was increased only in the small bowel, reaching its maximum in the ileum (Fig. 1a). Consequently, the presence of Firmicutes is constant along the intestine. Therefore, it is not unexpected that 13 OTUs of the *Lactobacillus* genus were found inside the core microbiota of all the samples (see Supplementary Fig. S1). The higher number of unique OTUs present in the duodenum was probably due to the microorganisms present in the regions from the mouth to the stomach, as well as those present in undigested food. Furthermore, possible explanations of why the microbiota uniquely present in the lower intestine does not appear in the upper intestine are the more rapid transit time in the small intestine^28^ and the different environmental conditions, such as oxygen concentrations^30,31^, which make the settlement and growth of certain microorganisms less likely.

Considering the correlations between genera inside each section, a tendency can be observed: the members of the Clostridiales order (*Clostridium* spp., *Sarcina* spp. and *SMB53* spp., amongst others) were always positively associated; however, their correlation with *Campylobacter* spp. differed depending on the region, positive in the duodenum and negative in the proximal colon. These findings could be explained by the oxygen concentration along the intestine, which decreases in the last intestine sections^28^. *Campylobacter* spp. are microaerophilic microorganisms, while most of the members of the Clostridiales order are obligate anaerobes^31^. In this context, the duodenum has a higher concentration of accessible oxygen^38^, and both microorganisms would take up their respective niches, not allowing the Clostridiales order to undergo excessive overgrowth, and thus, they would decrease competition with *Campylobacter* spp. for resources and space. Conversely, the reduced amount of oxygen in the colon regions favours anaerobic bacteria, such as those of the Clostridiales order, while the microaerophiles, with less oxygen available, will grow less. In this way, the Clostridiales order was also positively correlated with the sub-network formed by other anaerobic genera such as *Prevotella*^39^ and *Anaerovibrio*. Other genera such as *Lactobacillus*, which are facultative anaerobes^39^, can be present, as previously described, along the entire digestive tract and not be affected by oxygen competition. Finally, the opposition of *Prevotella* spp. with *Treponema* spp. found in the colon has been previously described in pigs by Ramayo-Caldas *et al*.^13^. Our guess is that these two hubs (*Prevotella* spp. and *Treponema* spp.), as well as the Clostridiales order sub-network, may compete for the degradation of dietary fibre^8,13,40^. Furthermore, the interactions between microorganisms may follow a series of universal dynamics, as has been recently proposed in the human gut microbiota^41^. However, this hypothesis needs to be validated in future studies involving larger sample sizes and adding more variables, such as age, sex, diet, and genetic background, amongst others, in order to study how the microbial interactions behave and change.

The functional prediction of the metagenome was in accordance with the microorganisms present in each intestine section and the differential abundances found in each comparison. In the first two comparisons, between the duodenum versus the jejunum and the jejunum versus the ileum, pathways related to photosynthesis were present, possibly due to the presence of the Cyanobacteria and of chloroplasts that were not yet digested inside the fodder. In the jejunum, the main microbiome functions were focused on extracting energy from carbohydrates, such as fructose and mannose, through glycolysis/gluconeogenesis. However, in the ileum, functions were related to fatty acid and pyruvate metabolism and xylene degradation, probably because of the abundance of Clostridiales^40^. Additionally, the sugar uptake functions predicted in this region were mainly associated with the phosphotransferase system. Therefore, if the genes associated with these functions are truly expressed, these findings would confirm a spatial organization of gut microbiota functions that allows the rapid internalization and conversion of the available simple carbohydrates for microbial proliferation and maintenance^42^.

SCFA production is increased in the large intestine^43^. However, in the comparison between the ileum and the proximal colon, the metabolism of two SCFAs (propanoate and butanoate) was more abundant in the ileum, probably due to the relative abundance of Clostridiales in this region (Fig. 1b). Nevertheless, other Clostridiales with butyric-acid activity, such as *Butyrivibrio* spp., were found in only the proximal colon.

In the last comparison, the proximal colon showed a higher abundance of *Prevotella* spp. than the distal colon. This last gut section had more OTUs belonging to the Ruminococcaceae family, which require anaerobic conditions and a carbohydrate energy source from dietary fibre, such as cellulose or xylan^44^. In accordance with the lower availability of oxygen in the large intestine, the energy pathways related to the citric acid cycle were dominant. These pathways are characteristic of the anaerobic bacteria mentioned above, such as *Prevotella* spp., which can degrade the proteins and polysaccharides in the plant cell wall, producing SCFAs^8^ that can be absorbed by the host^35^.

In our study, some Archaea related to methane metabolism were found to be differentially abundant in the hindgut, where methane is predominantly produced^43^. Moreover, the methane concentration increases towards the end of the intestine and can be modified depending on dietary fibre content^45^. Thus, methane metabolism was analysed in more detail (see Supplementary Fig. S2). In this figure, methanogenesis was more abundant in the distal colon, as expected. Furthermore, a higher abundance of acetate production in the hindgut than in the small intestine was found. Nonetheless, all these results must be considered carefully, because they are only predictions of the possible functions. Further studies regarding the metatranscriptome should be performed to clarify the real pathways that are present in these regions.

In summary, this study confirms that the energy pathways of the gut microbiome differ along its sections and represents, to the best of our knowledge, the first description of the gut microbiota composition along the intestine in Iberian pigs.

## Methods

### Ethics statement

The current study was performed according to the regulations of the Spanish Policy for Animal Protection RD53/2013, which complies with European Union Directive 2010/63/EU about the protection of animals used in experimentation. Pigs were housed in ITACyL animal facilities (Hontalbilla, Segovia, Spain), which meet local, national, and European requirements for Scientific Procedure Establishments. All experimental protocols were approved by the UCM (*Universidad Complutense de Madrid*) Ethics Committee, with reference number PROEX-007/15.

### Animals and lumen content collection

A convenience sample of thirteen 120-day-old Iberian castrated male pigs from the Torbiscal line was chosen as a compromise between the detection of relevant effects and the technical, ethical, and economical limitations of increasing the sampling size. In addition, a reduced variability in their microbiota composition was expected among animals raised under controlled environmental conditions and a uniform diet. Pigs were fed *ad libitum* with a standard fodder based on maize, wheat, barley, and soybean, with 3,320 kcal of digestible energy and 15.6% of crude protein. The pigs were slaughtered at an average weight of 48.7 kg. For each pig individually, its gastrointestinal tract was removed from the abdominal cavity and dissected immediately to collect the luminal content of each of the 5 sections less than 30 minutes after the pig’s death. The luminal contents of the 5 gut sections of each animal were gathered separately after isolating **~**10 cm of each section with 2 disposable adjustable plastic clamps on each side as follows: duodenum, first part after the stomach’s pyloric sphincter; jejunum, in the middle of the total small intestine’s length; ileum, last part of the small intestine; proximal colon, first part of the large intestine after the ileocaecal valve; distal colon, last part of the large intestine just before the rectum. Afterward, an incision in the middle of each section was performed with a scalpel under aseptic conditions. Finally, disposable sterile syringes with enlarged openings were used to collect a total of 8 mL of luminal content through the incision in each section and animal. The luminal contents were transferred to cryotubes, immediately frozen in liquid nitrogen, and later stored at −80 °C until used.

### DNA extraction and 16S rRNA gene sequencing

For each of the 65 samples (13 animals x five sections), the DNA of 0.2 g was extracted with the *PowerFecal*^®^ (*MoBio*^®^) kit, following the manufacturer’s recommendations, and DNA concentration and quality were measured with a *NanoDrop*^®^ Spectrophotometer ND-1000. The V3-V4 region of the 16S rRNA gene was amplified with two 16 S Amplicon PCR Primers (*Sigma-Aldrich*^®^): Forward, 5′ TCGTCGGCAGCGTCAGATGTGTATAAGAGACAGCCTACGGGNGGCWGCAG, and Reverse, 5′ GTCTCGTGGGCTCGGAGATGTGTATAAGAGACAGGACTACHVGGGTATCTAATC. These two primers were designed following the *Illumina*^®^ guide, *16S Metagenomic Sequencing Library Preparation*, based on the recommendations of Klindworth *et al*.^46^. The 65 PCR reactions were performed individually in a total volume of 25 μL using 12.5 ng of microbial DNA, 12.5 μL of 2× KAPA HiFi HotStart ReadyMix (*Kapa Biosystems*, Inc.) and 5 μL of each primer (1 μM) with the following program: 95 °C for 3 minutes, 25 cycles of three steps (95 °C for 30 s, 55 °C for 30 s and 72 °C for 30 s) and 72 °C for 5 minutes. The verification of the expected amplicon size (~550 bp) was done through agarose gel electrophoresis. Then, *AMPure*^®^ XP beads (*Beckman Coulter*, Inc.) were used to perform PCR product clean-up. The Nextera XT Index Kit was used to attach the dual indices, and another round of PCR clean-up was done with *AMPure*^®^ XP beads afterwards. Subsequently, the size (~630 bp) of the libraries from the indexed amplicons was validated with a DNA 1000 assay (*Agilent Technologies*, Inc) in a 2100 Bioanalyzer instrument (*Agilent Technologies*, Inc). Finally, the pooled libraries were sequenced in one run of a *MiSeq*^®^ (*Illumina*^®^) instrument in the SGB (*Servei de Genòmica i Bioinformàtica*, Cerdanyola del Vallès, Spain), using the *MiSeq*^®^ Reagent Kit v2 (500-cycle format, paired-end (PE) reads). A mean of 126,549 sequences for each sample was obtained.

### Taxonomic classification of the gut samples

The joining of the forward and reverse fastq files was performed with the *multiple_join_paired_ends.py* function in the QIIME pipeline^22^ (1.9.1. version). Following the recommendations by Bokulich *et al*.^47^ for raw data quality control and filtering steps, the sequences were filtered with a Phred score cut-off of 20 using the *split_libraries_fastq.py* command. Then, OTUs were identified with QIIME’s subsampled open-reference OTU calling approach, as proposed by Rideout *et al*.^48^, with the *pick_open_reference_otus.py* command and a subsampling percentage of 10% (s = 0.1). After this step, QIIME was utilized to identify and remove chimaeras with BLAST^49^. Lastly, the final OTU dataset was obtained by filtering out singletons and OTUs representing less than 0.005% of the total counts in each section^47^. In this sense, counts are defined as the number of sequences from each sample that hit the OTU clusters described in the Greengenes 13.8 taxonomic database^23^ or the “new OTU” clusters formed by QIIME.

### Diversity studies and differences in the abundances of the gut microbiota

The calculation of α and β-diversities as well as the NMDS were performed in R (www.r-project.org) through the vegan package^50^. In NMDS, the dissimilarity between pairs of samples was estimated with the Bray-Curtis distance^51^. The 5-part Venn diagrams were represented using the *draw.quintuple.venn* function of the VennDiagram R package^52^.

The OTU information table was merged at the genus level for each of the 65 samples through their available taxonomic information with the *tax_glom* method inside the phyloseq R package^53^. Then, the genera presence/absence analysis between each section and the next one was carried out with the metagenomeSeq R package^24^ using its *fitPA* function after filtering out the genera that were not present in either of the two compared sections. Thus, these genera, which were determined as not present in one of the two sections with an adjusted *p*-value ≤ 0.01 cut-off, were not considered for the genus-level differential abundance analysis. This second analysis was also performed with metagenomeSeq utilizing its *fitZig* function to create a model where the animal was included as a co-factor and only those genera with an adjusted *p*-value ≤ 0.01 cut-off were kept. Adjusted *p*-values for the *fitPA* and *fitZig* results were calculated in both cases through the false discovery rate (FDR) method^54^.

### Metagenome prediction and functional differences amongst gut sections

The metagenome KEGG^26^ orthologies (KOs) of the 65 samples were predicted with the PICRUSt software^25^, removing the OTUs that were not present in the Greengenes 13.5 database^23^. Then, the Bray-Curtis distance^51^ was used to measure the dissimilarity between pairs of samples to create an NMDS plot of the KOs with the vegan package^50^. After this step, the KOs were collapsed to the pathway level (KEGG level 3) with the *categorize_by_function.py* script of PICRUSt. The differences in abundance of these collapsed pathways were identified by the DESeq2 R package^27^ using a model where the animal was included as a co-factor. One pathway was considered more abundant in one section than the other when its adjusted *p*-value calculated through the FDR method^54^ was ≤ 0.01.

### Network prediction with the SPCIT approach

To infer the interaction patterns as well as the hub genera in each section, the network was calculated using the SPCIT method, as proposed by Ramayo-Caldas *et al*.^13^. First, to avoid the errors caused by the small number of samples in each section, the genera were split by section and filtered out if they represented less than 0.01% of the total section counts or they were not present (equals 0) in seven or more of the 13 samples. Then, due to violation of the sparsity assumption, the Sparse Correlations for Compositional data software (SparCC)^55^ advised application of the central log ratio transformation to the genus abundances in order to calculate correlations among the genera. To extract significant correlations, a strategy based on partial correlation and information theory was applied through the Partial Correlation coefficient with Information Theory (PCIT) algorithm^56^. The Cytoscape software^57^ was used to represent the network of partial correlations between the log-transformed genus abundances for each intestinal section. In the networks of the five intestinal sections, every node represents a genus, and every edge connecting two nodes represents a significant correlation. Following the recommendations by Ramayo-Caldas *et al*.^13^, from the pairwise correlation matrix obtained with SparCC in each section, median + 2 * SD was calculated as the cut-off. For clarity of presentation, only correlations above an absolute value of 0.65 were represented, as this value was the median cut-off of the five sections.

## Electronic supplementary material

Supplementary Information

Supplementary Table S1

Supplementary Table S1

Supplementary Table S3

## Data Availability

The raw sequencing data from this study were deposited in the NCBI Sequence Read Archive (SRA) under accession number SRP136308.
