# Supplementary material for: Characterization of bacterial microbiota compositions along the intestinal tract in pigs and their interactions and functions
Source: Sci Rep. 2018 Aug 24;8:12727. doi: 10.1038/s41598-018-30932-6 (PMC6109158; doi:10.1038/s41598-018-30932-6)
Supplement: Supplementary file 1 — Supplementary Information [file 41598_2018_30932_MOESM1_ESM.pdf]

Characterization of bacterial microbiota compositions along the intestinal tract in pigs and their interactions and functions

Daniel Crespo-Piazuelo[1,2,*], Jordi Estellé[3], Manuel Revilla[1,2], Lourdes Criado-Mesas[1], Yuliaxis Ramayo-Caldas[3,4], Cristina Óvilo[5], Ana I Fernández[5], Maria Ballester[4], and Josep M Folch[1,2]

[1] Plant and Animal Genomics, Centre for Research in Agricultural Genomics (CRAG), CSIC-IRTA-UAB-UB Consortium, Bellaterra, Spain

[2] Departament de Ciència Animal i dels Aliments, Facultat de Veterinària, Universitat Autònoma de Barcelona (UAB), Bellaterra, Spain

[3] Génétique Animale et Biologie Intégrative (GABI), Institut National de la Recherche Agronomique (INRA), AgroParisTech, Université Paris-Saclay, Jouy-en-Josas, France

[4] Departament de Genètica i Millora Animal, Institut de Recerca i Tecnologia Agroalimentàries (IRTA), Caldes de Montbui, Spain

[5] Departamento de Mejora Genética Animal, Instituto Nacional de Investigación y Tecnología Agraria y Alimentaria (INIA), Madrid, Spain

*E-mail: daniel.crespo@cragenomica.es

Supplementary information

Supplementary Tables

Supplementary Table S1. This table contains the results of the presence/absence analysis at the genus level performed with metagenomeSeq[24]. Each sheet represents one of the four consecutive correlations between the five sections for the 13 pigs. The colour shows which genus is uniquely present in that section when comparing the two sections: red, duodenum; yellow, jejunum; green, ileum; blue, proximal colon and purple, distal colon.

Supplementary Table S2. This table contains the results of the differential abundance analysis at the genus level performed with metagenomeSeq[24]. Each sheet represents one of the four consecutive correlations between the five sections for the 13 pigs. The colour shows which genus is more abundant in that section when comparing the two sections: red, duodenum; yellow, jejunum; green, ileum; blue, proximal colon and, purple, distal colon.

Supplementary Table S3. This table contains the results of the differential abundance analysis performed with DESeq2[27] for the KEGG[26] orthologies (KOs) predicted with PICRUSt[25] at the pathway level. Each sheet represents one of the four consecutive correlations between the five sections for the 13 pigs. The colour shows which pathway is more abundant in that section when comparing the two sections: red, duodenum; yellow, jejunum; green, ileum; blue, proximal colon and purple, distal colon.

Supplementary Figures

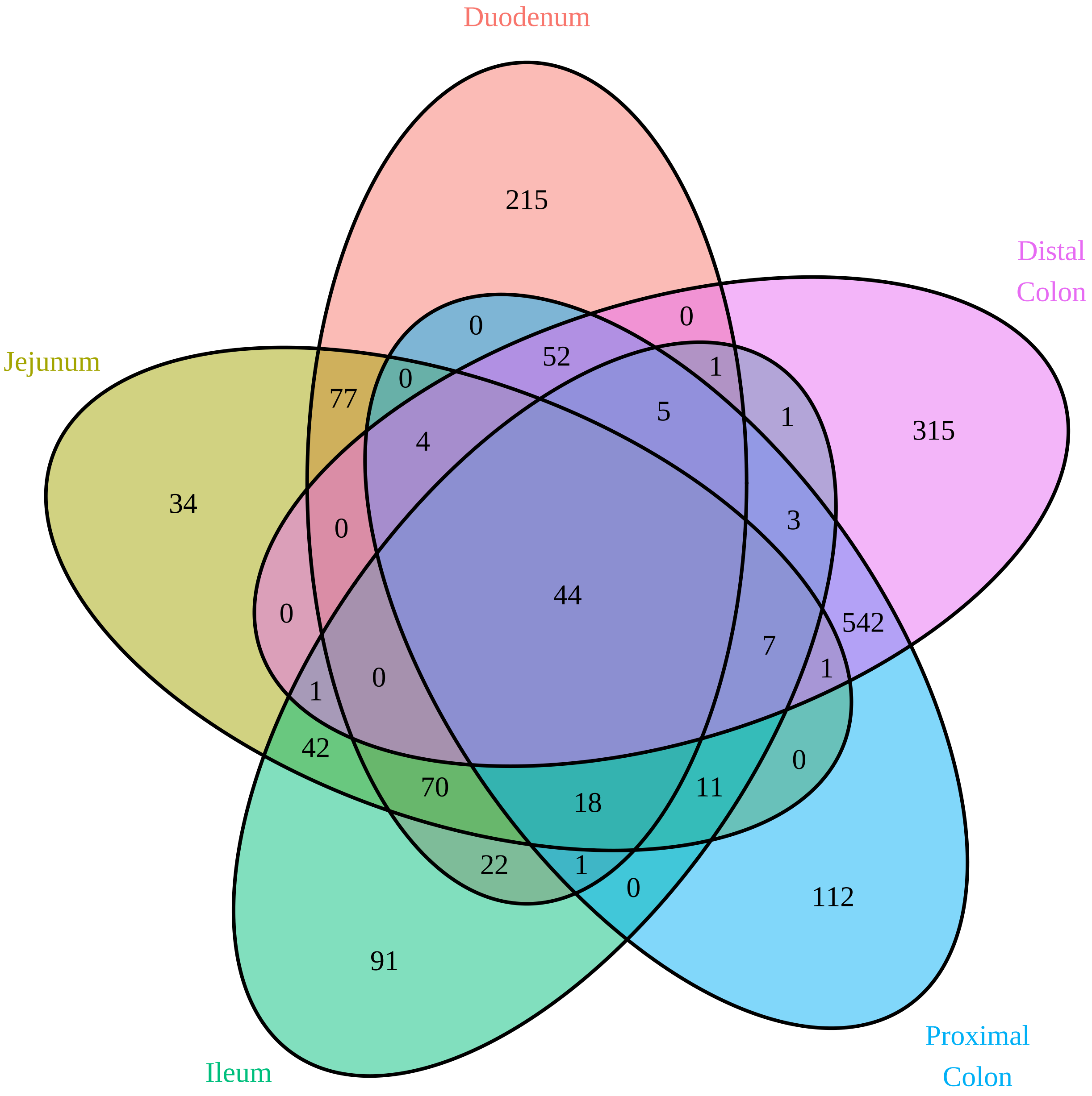

Supplementary Figure S1. Five-part Venn diagram performed for the OTUs shared among sections when combining the datasets from all subjects: duodenum (red), jejunum (yellow), ileum (green), proximal colon (blue), and distal colon (purple).

a)

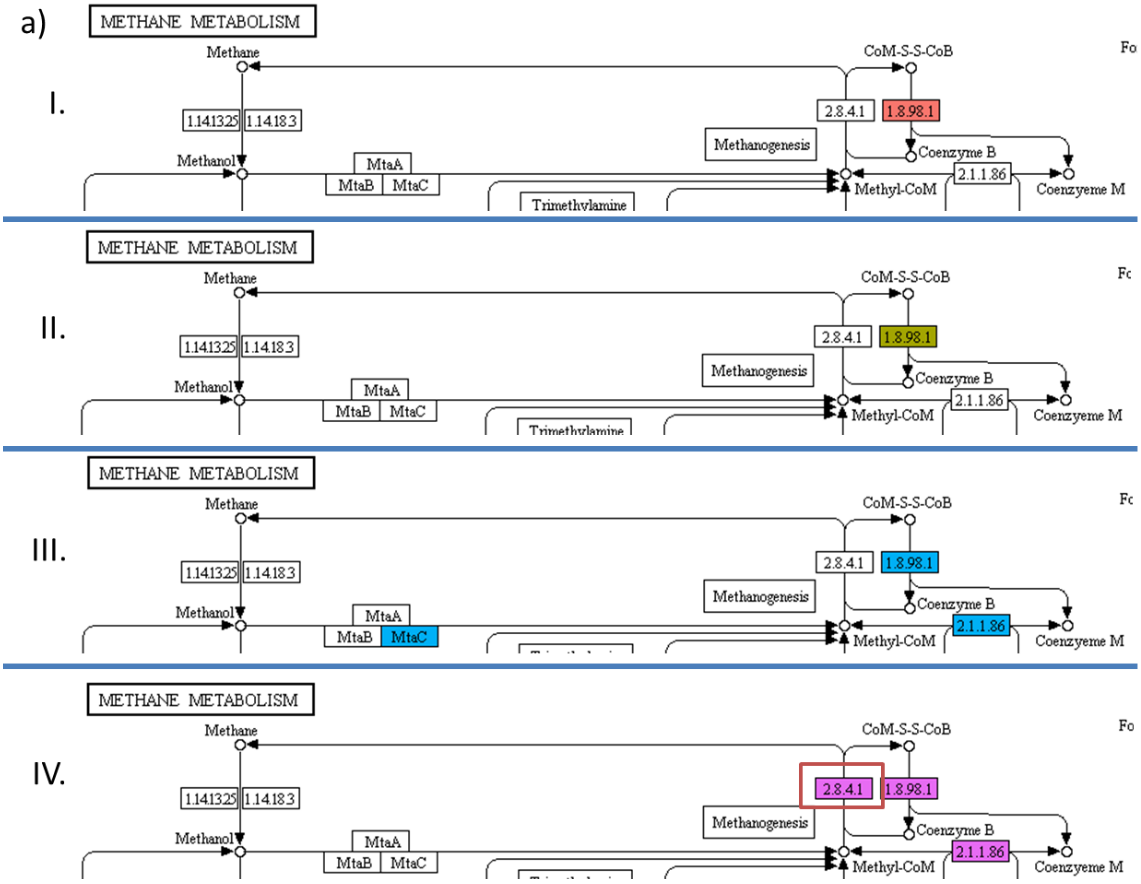
I.
II.
III.
IV.
METHANE METABOLISM
Methane
CoM-S-S-CoB
2.8.4.1
1.8.98.1
1.14.13.25
1.14.18.3
Methanogenesis
Coenzyme B
Methanol
MtaA
MtaB
MtaC
2.1.1.86
Methyl-CoM
Coenzyme M
Trimethylamine

b)

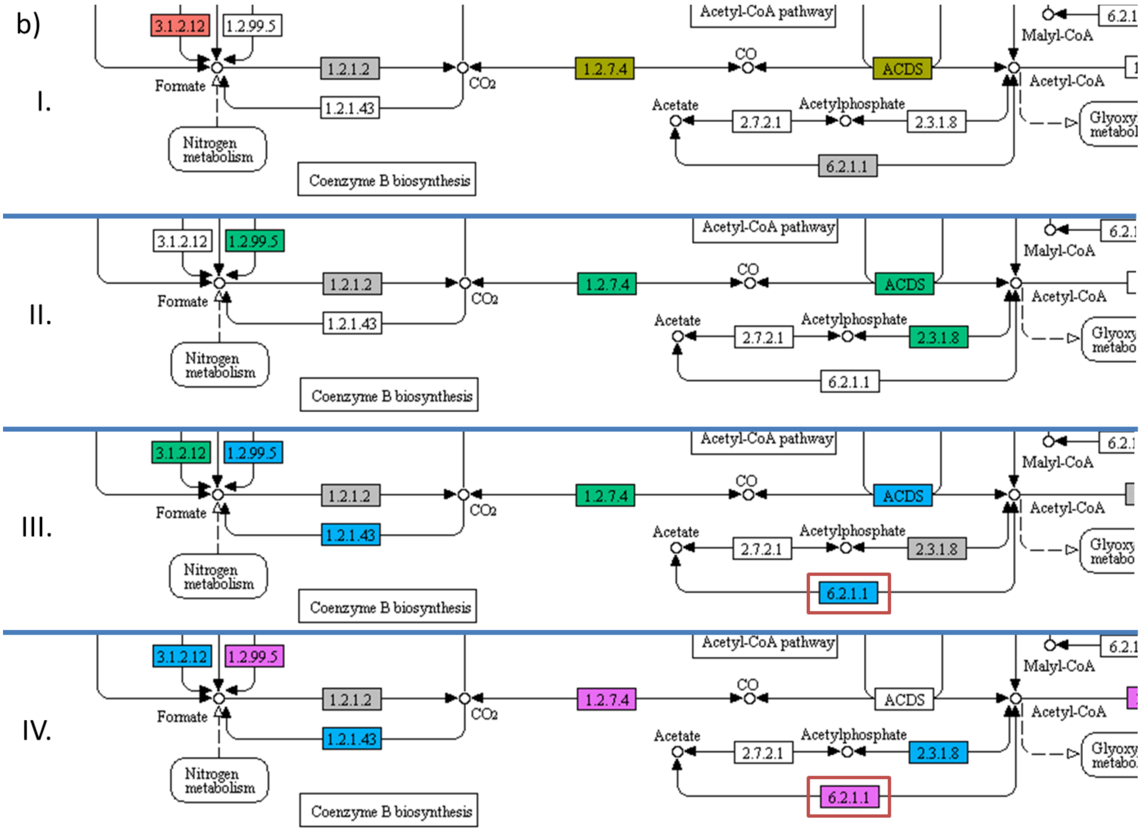
I.
II.
III.
IV.
3.1.2.12
1.2.99.5
Acetyl-CoA pathway
Formate
1.2.1.2
CO2
1.2.7.4
CO
ACDS
Malyl-CoA
Acetyl-CoA
1.2.1.43
Acetate
Acetylphosphate
2.7.2.1
2.3.1.8
Nitrogen metabolism
6.2.1.1
Coenzyme B biosynthesis

Supplementary Figure S2. DESeq2[27] results below a padj≤0.01 cut-off for the four comparisons between each pair of consecutive sections of the KEGG[26] orthologies (KOs) predicted by PICRUSt[25] represented over the KEGG[26] methane metabolism pathway (map00680): **I.** duodenum vs jejunum; **II.** jejunum vs ileum; **III.** ileum vs proximal colon; **IV.** proximal colon vs distal colon. The colour shows which KO was more abundant in that section when comparing the two sections: white, non-significant; red, duodenum; yellow, jejunum; green, ileum; blue, proximal colon and purple, distal colon. Ambiguous KOs were coloured grey. For clarity of presentation, the methane metabolism pathway was divided into two parts: **a)** The red rectangle shows how the production of methane was more abundant in the distal colon than in the rest of the comparisons. **b)** The two red rectangles represent how the production of acetate was more abundant in the proximal colon than in the ileum and more abundant in the distal colon than in the proximal colon.
